# Supplementary material for: A Global Screen for Assembly State Changes of the Mitotic Proteome by SEC-SWATH-MS
Source: Cell Syst. 2020 Feb 26;10(2):133–155.e6. doi: 10.1016/j.cels.2020.01.001 (PMC7042714; doi:10.1016/j.cels.2020.01.001)

O43414 | ERI3_HUMAN | ERI3 PINT1 PRNPIP PRNPIP1
Monomer MW [kDa]: 37.238 Monomer expected elution fraction: 49

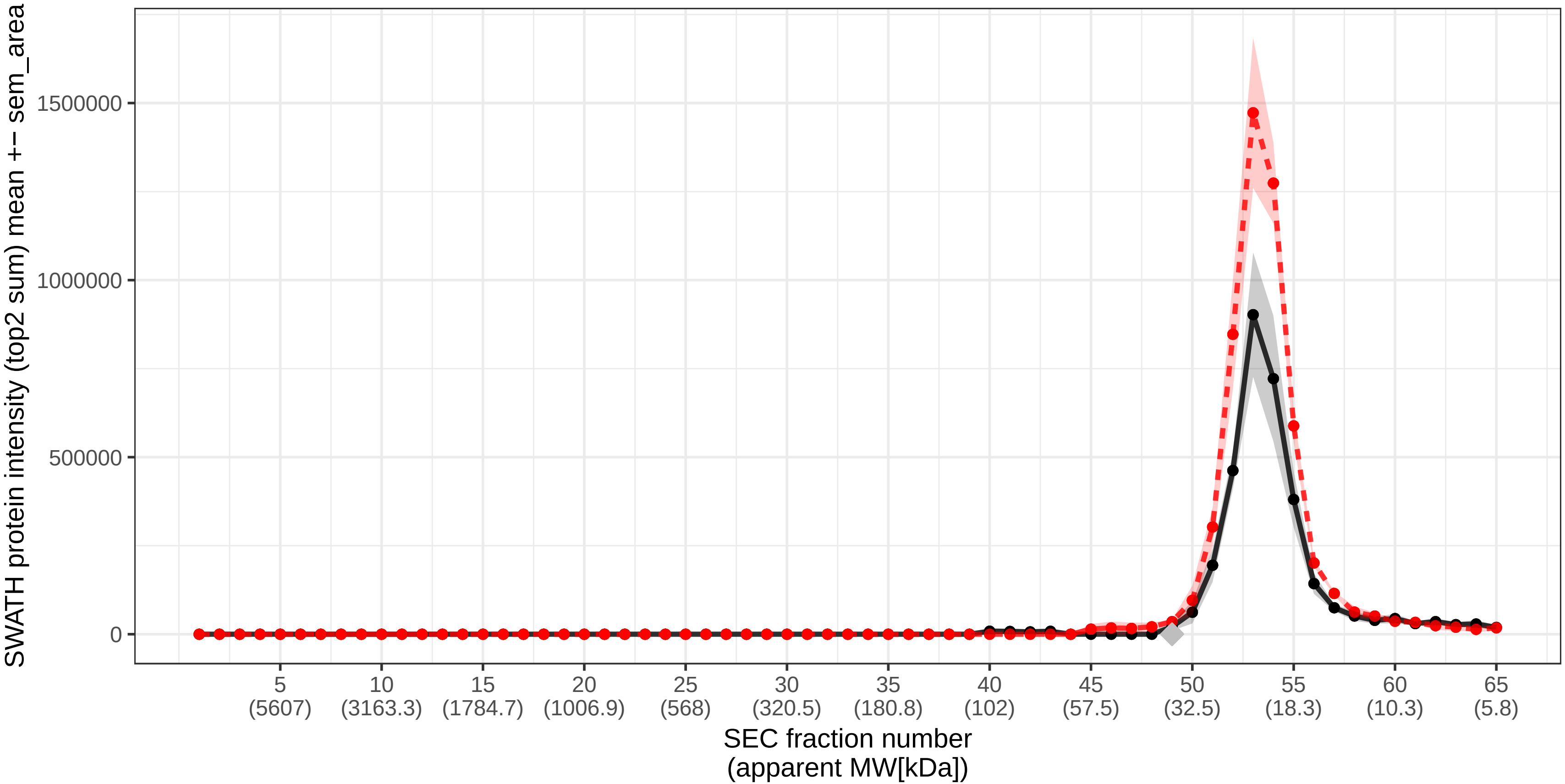

Supplement: Data S1. SEC-SWATH-MS Protein Chromatograms, Related to Figure 1 [file mmc6.zip › SECchrom_O43414_ERI3_HUMAN_ERI3_PINT1_PRNPIP_PRNPIP1.pdf]
